# Clinical characteristics and outcomes among Brazilian patients with severe acute respiratory syndrome coronavirus 2 infection: an observational retrospective study

**DOI:** 10.1590/1516-3180.2020.00365.R1.08092020

**Published:** 2020-11-27

**Authors:** Israel Júnior Borges do Nascimento, Luiz Ricardo Pinto, Valéria Alves Fernandes, Israel Molina Romero, João Antonio de Queiroz Oliveira, Milena Soriano Marcolino, Maria Fátima Leite

**Affiliations:** I PharmD. Clinical Pathologist and Medical Research Specialist, University Hospital and School of Medicine, Universidade Federal de Minas Gerais, Belo Horizonte (MG), Brazil; Medical Research Specialist, School of Medicine, Medical College of Wisconsin, Milwaukee, Wisconsin, United States.; II PhD. Full Professor, Department of Production Engineering, School of Engineering, Universidade Federal de Minas Gerais, Belo Horizonte (MG), Brazil.; III MSc, PhD. Researcher, University Hospital and School of Medicine, Universidade Federal de Minas Gerais, Belo Horizonte (MG), Brazil.; IV MD, PhD. Medical Researcher, Department of Infectious Diseases, Vall d'Hebron Hospital, Barcelona, Spain; Medical Researcher, Instituto Renê Rachou, Fiocruz Minas, Belo Horizonte (MG), Brazil.; V PharmB, MSc. Doctoral Student, University Hospital and School of Medicine, Universidade Federal de Minas Gerais, Belo Horizonte (MG), Brazil.; VI MD, PhD. Full Professor, University Hospital and School of Medicine, Universidade Federal de Minas Gerais, Belo Horizonte (MG), Brazil.; VII PharmD, PhD. Full Professor, Department of Physiology and Biophysics, Institute of Biological Sciences, Universidade Federal de Minas Gerais, Belo Horizonte (MG), Brazil.

**Keywords:** Severe acute respiratory syndrome coronavirus 2 [supplementary concept], COVID-19 [supplementary concept], Coronavirus infections, Mandatory reporting, Public reporting of healthcare data, Infectious diseases, nCoV-2019, Novel coronavirus, 2019-nCoV infections, 2019 novel coronavirus infection

## Abstract

**BACKGROUND:**

Since February 2020, data on the clinical features of patients infected by severe acute respiratory syndrome coronavirus 2 (SARS-CoV-2) and their clinical evolution have been gathered and intensively discussed, especially in countries with dramatic dissemination of this disease.

**OBJECTIVE:**

To assess the clinical features of Brazilian patients with SARS-CoV-2 and analyze its local epidemiological features.

**DESIGN AND SETTING:**

Observational retrospective study conducted using data from an official electronic platform for recording confirmed SARS-CoV-2 cases.

**METHODS:**

We extracted data from patients based in the state of Pernambuco who were registered on the platform of the Center for Strategic Health Surveillance Information, between February 26 and May 25, 2020. Clinical signs/symptoms, case evolution over time, distribution of confirmed, recovered and fatal cases and relationship between age group and gender were assessed.

**RESULTS:**

We included 28,854 patients who were positive for SARS-CoV-2 (56.13% females), of median age 44.18 years. SARS-CoV-2 infection was most frequent among adults aged 30-39 years. Among cases that progressed to death, the most frequent age range was 70-79 years. Overall, the mortality rate in the cohort was 8.06%; recovery rate, 30.7%; and hospital admission rate (up to the end of follow-up), 17.3%. The average length of time between symptom onset and death was 10.3 days. The most commonly reported symptoms were coughing (42.39%), fever (38.03%) and dyspnea/respiratory distress with oxygen saturation < 95% (30.98%).

**CONCLUSION:**

Coughing, fever and dyspnea/respiratory distress with oxygen saturation < 95% were the commonest symptoms. The case-fatality rate was 8.06% and the hospitalization rate, 17.3%.

## INTRODUCTION

The ongoing pandemic caused by the novel severe acute respiratory syndrome coronavirus 2 (SARS-CoV-2) has been associated with a greater number of deaths occurring more rapidly than had been observed among previously leading causes of mortality, such as unintentional injuries, stroke and heart diseases. As of July 6, 2020, more than 11,495,412 confirmed cases have been reported, along with more than 535,185 officially notified deaths.^1^ In developing countries, specific data regarding incidence, local clinical manifestations, radiological and laboratory abnormalities and requirements for establishment of differential diagnoses considering local peculiarities still remain obscure and are often insufficient. In Brazil, as of June 15, 2020, 1,603,055 cases and 64,867 deaths had been legally counted.^1^

So far, according to studies conducted in developed countries, the typical signs and symptoms of the novel 2019 coronavirus are fever, coughing (with or without sputum), sore throat, and shortness of breath (with or without associated respiratory distress comprising oxygen saturation < 95.0%).^2^^,^^3^ However, new symptomatic profiles are being described in the literature, almost on a daily basis. Manifestations such as acute olfactory disorders, acute hyposmia and anosmia, dysgeusia and dermatological complaints might also be present with the onset of coronavirus disease (COVID-19).^4^^–^^7^

Although several studies have already described symptom profiles for patients in European and Asian-Pacific countries, at present there is no study providing detailed information within the Brazilian populational setting. Indeed, few papers on COVID-19 symptom profiles have been published in developing and poor or middle-income countries. Additionally, the studies relating to the settings of developing countries have been either case series, typically with fewer than 100 patients, or case reports, which do not necessarily describe the real epidemiological status of either low or middle-income countries.^8^^–^^10^ None of these studies assessed the most common clinical presentations of the novel coronavirus in Brazilian patients. Nor did they attempt to investigate differences in clinical presentation or underlying diseases among patients infected with this novel virus.

## OBJECTIVE

In this study, we aimed to assess the clinical features of Brazilian patients infected with SARS-CoV-2 and to analyze patient mortality and the need for hospital admission.

## METHODS

### Study design

This was an observational retrospective study, based on individual data from Brazilian patients that were collected from the Center for Strategic Health Surveillance Information of the Health Secretariat of the Brazilian Ministry of Health. This government branch targets early detection through establishing continuous monitoring, in order to deliver adequate solutions for public healthcare emergencies such as COVID-19. Ethical approval was obtained from a local ethics committee (reference number 30350820.5.0000.0008), which was granted on April 13, 2020. The study authors did not have any contact with the patients described here, and did not deliver any pharmacological or non-pharmacological intervention to them.

### Settings

All the confirmed patients included in this study were admitted to primary care centers, private clinics or hospital facilities in the state of Pernambuco, in northeastern Brazil. According to official governmental reports, as of June 10, 2020, Pernambuco had the seventh largest number of confirmed cases in Brazil (41,010 accumulated cases).^11^ Overall, with a total area of 98,311 square kilometers, Pernambuco has around 8.8 million inhabitants, and in 2017 was considered to have a medium human development index (0.67).^12^^,^^13^ However, because of regional discrepancies within this state regarding access to education, life expectancy and per capita income, it should be noted that several municipalities in the state have low human development indexes (< 0.50).

### Participants

All patients, regardless age, who presented to any healthcare facility (public or private) with suspected SARS-CoV-2 infection between February 26 and May 25, 2020, and who were registered on the government's online platform for suspected cases were eligible for inclusion in this study. At the platform interface, patients are enrolled as “suspected cases – under investigation” and as soon the laboratory result has been shared with the requesting healthcare center, the designated medical provider can update the patients' status to “negative for SARS-CoV-2 infection” or “positive for SARS-CoV-2 infection”, based on the report from the real-time quantitative polymerase chain reaction (RT-qPCR). There is also the possibility of providing results relating to alternative causes of infection that might be investigated (such as influenza A or B).

In our study, only patients with confirmed SARS-CoV-2 were included in the descriptive analysis. Therefore, patients were excluded if their laboratory result was negative for SARS-CoV-2. All the infected patients included in the present study had tested positive for SARS-CoV-2 through use of RT-qPCR on samples from nasopharyngeal or oropharyngeal specimens. The eligibility criterion for a positive diagnosis of SARS-CoV-2 infection was that at least one gene region was recognized and amplified as positive for viral proteins (nucleocapsid and open reading frame).

Informed consent was not required because we used secondary data from an official database. The RT-qPCR assay was performed either in the Central Public Health Laboratory (LACEN) or in private diagnostic laboratories.

### Variables and outcomes

The main primary variable of the study was clinical manifestation of SARS-CoV-2 infection among the patients, along with categorization of these patients according to the outcome at the end of the follow-up period (i.e. on May 25, 2020). Thus, the patients who had been enrolled were classified into five outcome groups. Patients with a definitive clinical status were stratified as “Recovered” (patients who after medical assessment were considered not to present active infection) or “Died” (patients who progressed to death) and were compared with each other. Similarly, individuals with a transient clinical status (i.e. awaiting case improvement or worsening) were categorized as “Domestic quarantine” (patients who had been directed to place themselves in isolation at home), “Admitted to hospital care” (patients who, on May 25, 2020, were in a hospital, either in an isolation ward or in an ordinary hospital bed) or “Admitted to intensive care unit (ICU)” (patients who, on May 25, 2020, were hospitalized in an ICU).

Exploratory variables such as the case distribution according to age group and gender, temporal distribution of included cases, time elapsed between notification and death and time elapsed between symptom onset and death were also analyzed.

### Data sources and measurements

In Brazil, a country with both single and multi-payer systems (public and private healthcare systems, respectively), notification of all confirmed SARS-CoV-2 cases (clinically classified as influenza-like syndrome or severe acute respiratory syndrome) has become mandatory since March 2020. These cases are registered in online servers and the records are subsequently processed. The notification and data registration are performed by healthcare personnel and once the laboratory result has been disclosed to the medical facility, the designated medical provider can update the diagnosis status in the system.

Influenza-like illness is defined as febrile sensation or fever, associated with coughing or sore throat or running nose or shortness of breath. Severe acute respiratory syndrome is defined as influenza-like symptoms with dyspnea/respiratory distress or persistent thoracic pressure or oxygen saturation < 95% in ambient air or peripheral cyanosis.

For non-hospitalized patients, such as patients attended in the primary care sector or at private clinics, the “e-SUS VE” is the final online host system for all suspected cases. On the other hand, cases of severe acute respiratory syndrome and deaths need to be notified through the Information System for Influenza Epidemiological Surveillance (Sistema de Informação de Vigilância Epidemiológica da Gripe, SIVEP-Gripe). In the state of Pernambuco, which is potentially the most transparent state in Brazil with regard to data sharing and epidemiological surveillance, reports from both systems are periodically integrated and compiled into a single online platform.^13^

All data associated with clinical symptoms and signs, previous health history and epidemiological features were extracted from the electronic panel of cases of novel coronavirus infection in the state of Pernambuco, Brazil. Two experienced medical research specialists reviewed and abstracted the data. After initial processing, the data were entered into a computerized database (Microsoft SQL Server, version 2019, United States) and were cross-checked.

### Study size and statistics

No formal sample size calculation was carried out, because of the observational and convenience-sampling nature of the study. The statistical evaluation included descriptive analysis on the study population and comparisons between groups using the chi-square test. We defined differences as statistically significant if the P-value was < 0.05. Categorical variables were expressed as the number and its respective percentage. The Statistical Package for the Social Sciences (SPSS), version 20.0 (IBM, New York, United States) was used to obtain mathematical evaluations.

## RESULTS

### Participants' characteristics

Overall, the cases of 54,235 patients were retrieved from the governmental database up to May 25, 2020. Of these, 28,854 patients had a confirmed laboratory diagnosis of SARS-CoV-2, 22,034 were negative for virus detection and 3,347 were waiting for laboratory results.

In the study sample (infected patients; n = 28,854), the median age was 44.18 years and 56.13% were female, with a male to female ratio of 0.78. The largest proportion of the infected patients was aged between 30 and 39 years (n = 6,949; 24.08%). Information on underlying diseases was not reported for all patients, and it was not possible to know which patients did not have underlying diseases, or in which cases some variables were missing. Among the patients with any descriptions of preexisting comorbidities, hypertension (n = 863), diabetes (n = 533), obesity (n = 110), chronic renal failure (n = 90), history of stroke (n = 85) and asthma (n = 63) were the most prevalent ones. Among all the patients included, 22 (0.07%) were classified as having an additional ongoing viral coinfection (either influenza A or influenza B) at the time of the notification.

### Descriptive data

After distribution of the patients into definitive outcomes, 8,863 patients (30.7%) were considered to have recovered of the infection, while 2,328 (8.06%) died due to complications from the infection. Male patients were more likely to progress to death (55.0%) (Table 1). For both genders, the majority of fatalities occurred in the group of patients older than 60 years. Among females, the majority of deaths were among individuals older than 80 years, while among male individuals, patients aged between 60 and 69 years progressed to death more frequently. Female patients recovered more frequently than did males (62.63%).

**Table 1:** Age distribution among the patients described in the data retrieved

Age groups	Confirmed cases (n = 28,854) n (%)		Recovered cases (n = 8,863) n (%)		Case-fatalities (deaths) (n = 2,328) n (%)	
	Female	Male	Female	Male	Female	Male
0 to 9 years	444 (1.53)	373 (1.29)	156 (1.76)	140 (1.56)	6 (0.25)	5 (0.21)
10 to 19 years	178 (0.61)	143 (0.49)	42 (0.47)	36 (0.40)	4 (0.17)	4 (0.17)
20 to 29 years	2,018 (6.99)	1,262 (4.36)	764 (8.62)	408 (4.60)	14 (0.60)	15 (0.64)
30 to 39 years	4,171 (14.45)	2,778 (9.62)	1,768 (19.95)	926 (10.43)	24 (1.03)	54 (2.32)
40 to 49 years	3,717 (12.88)	2,861 (9.91)	1,509 (17.02)	882 (9.94)	60 (2.57)	127 (5.46)
50 to 59 years	2,552 (8.84)	2,098 (7.26)	886 (9.99)	546 (6.15)	150 (6.44)	179 (7.69)
60 to 69 years	1,325 (4.59)	1,376 (4.75)	237 (2.68)	208 (2.34)	246 (10.57)	315 (13.54)
70 to 79 years	947 (3.28)	1,059 (3.66)	102 (1.16)	98 (1.10)	258 (11.09)	312 (13.40)
> 80 years	846 (2.93)	706 (2.43)	87 (0.98)	68 (0.75)	286 (12.29)	269 (11.56)
**Total**	**16,198 (56.13)**	**12,656 (43.87)**	**5,551 (62.63)**	**3,312 (37.37)**	**1,048 (45.01)**	**1,280 (54.99)**

During the analyzed period, there were 22 confirmed co-infection of influenza A or influenza B. In addition, there were 54,235 cases registered in the database (including suspected, confirmed and negative cases).

Regarding transient outcomes, 4,771 individuals (16.5%) were admitted to an isolation ward, 1,442 (5.0%) were directed to place themselves in domestic quarantine and 227 (0.78%) were hospitalized in an intensive care unit. For 10,996 patients with confirmed SARS-CoV-2 infection (38%), the final outcome was not available or not declared.

In the overall cohort of confirmed patients, the median length of time from symptom onset to regulatory notification was 7.0 days (interquartile range, IQR 4.0-10.0). Among patients who progressed to death, the median length of time between symptom onset and notification was 5.0 days (IQR 3.0-8.0), while the median length of time between symptom onset and death was 8.0 days (IQR 5.0-14.0).

### Analysis on the clinical characteristics of confirmed cases of SARS-CoV-2

A summary of the clinical manifestations of the 28,854 confirmed cases of SARS-CoV-2 infection is shown in Table 2. Overall, signs or symptoms of some type were registered in relation to 17,631 patients (61.10%). Thus, notifications were made in the cases of 38.9% (n = 11,223) of the confirmed patients and these cases were registered in the database. However, none of the clinical information was precisely inserted.

**Table 2:** Clinical data from confirmed cases of SARS-CoV-2 infection in the state of Pernambuco, Brazil (data up to May 25, 2020)

Clinical presentation of confirmed patients (n = 17,631)		Domestic quarantine (n = 1,442) n (%)	Recovered (n = 8,863) n (%)	Case-fatalities (n = 2,328) n (%)	Admitted to isolation ward (n = 4,771) n (%)	Admitted to ICU (n = 227) n (%)	Comparison between recovered and case-fatality patients (P-value)	Comparison between patients admitted to isolation ward and ICU (P-value)
Coughing (n = 12,232)		874 (0.60)	6,124 (0.69)	1,574 (0.67)	3,512 (0.73)	148 (0.65)	0.169	0.005
Fever (n = 10,976)		853 (0.59)	5,256 (0.59)	1,493 (0.64)	3,236 (0.67)	138 (0.60)	< 0.001	0.027
Dyspnea or respiratory distress with SpO_2_ < 95% (n = 8,941)		507 (0.35)	2,619 (0.29)	2,057 (0.88)	3,559 (0.74)	199 (0.87)	< 0.001	< 0.001
Sore throat or odynophagia (n = 4,847)		460 (0.31)	3,470 (0.39)	216 (0.09)	664 (0.13)	37 (0.16)	< 0.001	0.313
Myalgia (n = 1,416)		460 (0.31)	333 (0.03)	99 (0.04)	513 (0.10)	11 (0.04)	0.270	0.005
Vomiting or nausea or diarrhea (n = 1,293)		186 (0.12)	316 (0.03)	191 (0.08)	582 (0.12)	18 (0.07)	< 0.001	0.053
Headache (n = 1,049)		277 (0.19)	352 (0.03)	51 (0.02)	361 (0.07)	8 (0.03)	< 0.001	0.023
Anosmia (n = 801)		223 (0.15)	284 (0.03)	31 (0.01)	257 (0.05)	6 (0.02)	< 0.001	0.070
Adynamia or asthenia (n = 545)		55 (0.03)	120 (0.01)	77 (0.02)	287 (0.06)	6 (0.02)	< 0.001	0.035
Dysgeusia or loss of taste (n = 490)		170 (0.11)	218 (0.02)	20 (< 0.01)	77 (0.01)	5 (0.02)	< 0.001	0.495
Hyporexia (n = 138)		10 (< 0.01)	10 (< 0.01)	31 (0.01)	85 (0.01)	2 (< 0.01)	< 0.001	0.311
Abdominal pain (n = 88)		7 (< 0.01)	13 (< 0.01)	19 (< 0.01)	48 (0.01)	1 (< 0.01)	< 0.001	0.398
Sneezing (n = 46)		9 (< 0.01)	27 (< 0.01)	1 (< 0.01)	9 (< 0.01)	–	0.025	0.512
Eye pain (n = 20)		5 (< 0.01)	16 (< 0.01)	2 (< 0.01)	3 (< 0.01)	–	0.724	0.705
Chest pain (n = 12)		3 (< 0.01)	4 (< 0.01)	1 (< 0.01)	4 (< 0.01)	–	0.965	0.663
Running nose (n = 9)		4 (< 0.01)	154 (0.01)	19 (< 0.01)	2 (< 0.01)	–	< 0.001	0.758
Asymptomatic (n = 9)		1 (< 0.01)	5 (< 0.01)	1 (< 0.01)	2 (< 0.01)	–	0.803	0.758
Not declared or not available (n = 1,339)		279 (0.19)	704 (0.07)	107 (0.04)	239 (0.05)	10 (0.04)	< 0.001	0.683
**Comorbidities**								
	Hypertension (n = 863)	17 (0.01)	63 (< 0.01)	643 (0.27)	126 (0.02)	14 (0.06)	< 0.001	0.002
	Diabetes (n = 533)	1 (< 0.01)	182 (0.02)	309 (0.13)	35 (< 0.01)	6 (0.02)	< 0.001	0.002
	Obesity (BMI > 25 kg/m^2^) (n = 110)	1 (< 0.01)	6 (< 0.01)	86 (0.03)	14 (< 0.01)	3 (0.01)	< 0.001	0.009
	Chronic renal failure (any stage) (n = 90)	–	2 (< 0.01)	76 (0.03)	11 (< 0.01)	1 (< 0.01)	< 0.001	0.528
	History of stroke (n = 85)	–	2 (< 0.01)	72 (0.03)	10 (< 0.01)	1 (< 0.01)	< 0.001	0.468
	Asthma (n = 63)	–	12 (< 0.01)	35 (0.01)	14 (< 0.01)	2 (< 0.01)	< 0.001	0.126
	Chronic obstructive pulmonary disease (n = 48)	–	5 (< 0.01)	36 (0.01)	4 (< 0.01)	3 (0.01)	< 0.001	< 0.001
	Any neoplasia (n = 33)	–	3 (< 0.01)	26 (0.01)	4 (< 0.01)	–	< 0.001	0.663
	History of myocardial infarction (n = 32)	–	1 (< 0.01)	27 (0.01)	3 (< 0.01)	1 (< 0.01)	< 0.001	0.049
	Chronic liver disease or hepatitis (n = 11)	–	1 (< 0.01)	5 (< 0.01)	5 (< 0.01)	–	< 0.001	0.626
	HIV infection (under control or not) (n = 9)	–	3 (< 0.01)	4 (< 0.01)	2 (< 0.01)	–	0.018	0.758
	Transplanted (n = 3)	–	–	2 (< 0.01)	1 (< 0.01)	–	0.006	0.827
	Alcoholism (n = 1)	–	–	1 (< 0.01)	–	–	0.051	–
	Without comorbidities or not declared or not available (n = 8,308)	866 (0.60)	3,640 (0.41)	767 (0.32)	2,882 (0.60)	153 (0.67)	< 0.001	0.035

ICU = intensive care unit; BMI = body mass index; HIV = human immunodeficiency virus.

It is important to state that the comparison shown above relates to: 1) Comparison between patients who progressed to death (case-fatalities) and patients who recovered; and 2) Comparison between patients who were admitted to an isolation ward and those admitted to an ICU. Therefore, for each symptom and comorbidity category, we performed statistical analysis to check whether there was any group-to-group significant difference.

The main clinical manifestations observed among the patients comprised coughing (with or without sputum) (42.39%), fever (38.03%), dyspnea or respiratory distress with oxygen saturation lower than 95% (30.98%), sore throat or odynophagia (16.79%), myalgia (4.90%) and headache (3.63%). Less common symptoms such as anosmia (2.77%), adynamia or asthenia (1.88%), dysgeusia or loss of taste (1.6%) and hyporexia (0.047%) were also reported.

Comparison between the patients who recovered and those who died showed that dyspnea or respiratory distress with oxygen saturation < 95% (29.0% versus 88.0%) and fever (59.0% versus 64.0%) were significantly more frequent among the patients who died. Sore throat was more frequent among the patients who recovered (39.0% versus 9.0%). Comparison between patients hospitalized in an isolation ward and patients in an ICU showed that fever (67.0 versus 60.0%) was significantly more frequently observed among the patients in an isolation ward. Among the patients admitted to an ICU, there was higher frequency of manifestation of dyspnea than among those in an isolation ward (74.0 versus 87.0%).

Hypertension, diabetes and obesity were more frequently reported among patients admitted to an ICU and among the patients who died. A complete description of underlying diseases observed among the patients included, along with comparisons between patients who progressed to death (case-fatalities) and patients who recovered and between patients who were admitted to an isolation ward and those who were admitted to an ICU, for each symptom and comorbidity, is shown in **Table 2**.

## DISCUSSION

Over the last few weeks, Brazil has become the epicenter of the novel coronavirus pandemic.^14^^–^^16^ With the global impact of the novel coronavirus, it is important to highlight that different populations can manifest different clinical symptoms and can progress differently over the natural course of the infection. Overall, the most commonly reported clinical features consisted of coughing (with or without sputum) (42.39%), fever (38.03%) and dyspnea or respiratory distress with oxygen saturation lower than 95% (30.44%).

Our results showed slightly lower prevalences for most observed clinical features and comorbidities, compared with previous studies.^3^ Severe illness (defined as patients requiring hospitalization) occurred in 17.3% of the patients. Indeed, fever and dyspnea were remarkably more frequently reported among fatalities. In addition, dyspnea and oxygen saturation < 95% were shown to be contributing factors for admission to an ICU.

With regard to underlying diseases, the comorbidities most often registered were hypertension, diabetes, obesity and chronic renal failure. Additionally, taking into account underlying pathological conditions, we observed that there was an association between the presence of comorbidities and worse progression of the disease. Regarding coexistence of underlying conditions, we perceived that the frequency of comorbidities was slightly lower among the cases reported here than in previously published data.^2^ However, this may have been mainly caused by the singularities of the hospital environment and the features of the emergency department. In emergency departments, it is very frequently impossible to obtain a detailed medical history.

Even though disease profiling for COVID-19 has been replicated and implemented in several countries, this was the first study to describe its main clinical characteristics and outcome distribution in Brazil using a substantial number of patients. Brazil is a country with continental geographical proportions and has a wide spectrum of tropical infectious diseases (most of them neglected), such as Chagas disease, leishmaniasis and dengue. However, to date, no previous diseases has had the impact of abruptly increasing the number of patients seeking medical consultations.^17^ In association with Brazil's large territorial proportions, it is also a country with social and economic inequalities, which consequently influences the health status of its inhabitants.^18^ Thus, as the novel coronavirus has disseminated across the country, the impact of the disease on low-income populations has been increasing substantially, thus resulting in serious negative effects among these citizens.

The extensive spectrum of reported symptoms during admission (with several body systems involved), together with the wide range of severity (from asymptomatic cases to severely ill patients), may potentially cause an initial misdiagnosis, especially for patients whose first RT-qPCR is negative.^4^ We found that the frequency of reports on anosmia/hyposmia and other minor symptoms as dermatological manifestations was low. However, considering that the reporting of these symptoms only started in mid-April, medical care for these manifestations in our cohort within a Brazilian setting may have been delayed or been given less attention. Nevertheless, several studies have already reported that these particular symptoms are highly sensitive for diagnosing the disease.^4^ In addition, developing countries like those in Latin America and Africa have their own endemic diseases that are currently presenting increasing incidence. This increases the challenge involved in reaching a conclusive final diagnostic hypothesis.^19^

Fever was more prevalent among the patients who died than among those who required hospital admission. However, we hypothesized that this may have been due to lack of completion of the reporting questionnaire. Patients who needed hospital care may have less frequently filled out the entire questionnaire.

In our study, the majority of the symptoms were associated with alternative infections, such as influenza, rhinovirus, dengue fever or gastroenteritis. Therefore, we highlight the fact that in areas in which concomitant outbreaks may have been occurring in parallel, use of differential diagnosis should always be borne in mind. Through this, presence of potential secondary pathogens can be ruled out and clinical management of greater accuracy can be implemented for patients for whom a differential diagnosis could not yet be established.

In our study, 8% (n = 2,328) of the patients with SARS-CoV-2 infection progressed to death (in less than three months). The mortality rate in the state of Pernambuco was also slightly higher than the Brazilian national average, possibly because of the economic peculiarities of the region and because of lack of hospital infrastructure for severe cases.^20^ In addition, the explanations for this higher mortality rate may relate to delayed diagnosis of the disease, fundamentally caused by limitations on the availability of laboratory tests and trained medical personnel. The explanations may even relate to patients' fear of seeking medical care in the early stage of the disease. This would consequently favor greater severity of clinical condition at the time of late hospital admission.

Furthermore, in the state of Pernambuco, a significant number of municipalities face either geographical or structural difficulties with regard to accessing appropriate medical treatment. One compelling example of these challenges is that, by the end of the period analyzed (May 25, 2020), 98% of the beds available for COVID-19 patients (in isolation wards or ICUs) were occupied. Thus, especially in settings where social and economic discrepancies prevail, this disease is of extreme importance, considering its social, economic or public health-related impacts.

Although the total number of individuals infected with SARS-CoV-2 may have been underestimated, this disease is an important public healthcare issue in Brazil and in developing countries across the globe. Taking into account the entire year of 2018 (when the total number of deaths in the state of Pernambuco was 62,011), the current number of fatalities resulting from SARS-CoV-2-related infections corresponds to the same mortality rate for all other infectious diseases aggregated (including flu, tuberculosis, all forms of hepatitis and HIV).^20^ In relation to the body of literature, the mortality rate observed in our study was slightly higher than rates seen in other settings such as China and Italy.^20^^–^^23^

Our data suggested that the mortality rate among male Brazilian subjects was higher than the rate among females. This had also been observed in previous studies.^3^ Even though it was perceived that female patients accounted for 56.0% of the total number of confirmed cases of infection, there was a higher mortality rate among male patients (55.0%).

There are different hypotheses to explain this fact. Initially, it was suggested that women might be less susceptible to viral infections than men due to higher production of circulating antibodies along with prolonged levels of these biomarkers.^22^^,^^23^ Additionally, another factor that might explain the lower susceptibility of female patients to the novel coronavirus infection is their production of estrogen and immune factors linked to X chromosomes.^24^ In women, the double X chromosome affects the immune system with regard to expression of several elements, such as the expression of toll-like receptor 7 (TLR7).^25^ Since TLRs are expressed at higher levels in women and their expression leads to higher immune responses, it has been suggested that these two associated factors might therefore increase resistance to viral infections. Another cell-related explanation for the higher immunoprotection among female patients than among male patients relates to CD4+ T cells.^26^ Expression of these cells is higher in women and, thus, a state of higher immune response may be achieved in females than in males, which also would provide a more protected status.^25^^,^^26^ Lastly, but not least, cultural features can also account for the imbalanced mortality rate between male and female patients.

In Brazil, promotion of healthcare policies for women has brought this population closer to healthcare facilities, both for elective medical procedures and for emergencies.^27^^,^^28^ In addition, especially in traditional areas like northeastern Brazil, the stereotype of the masculine image, depicted as the family progenitor who never gets sick, can also be related to this sociocultural feature.^20^^,^^30^ Thus, even with the observed disparity of confirmed cases between males and females, male patients are at higher risk of a fatal outcome than are female patients.

The main strength of the current study was that laboratory data on more than 28,000 confirmed cases were examined. Patients who only had a clinical diagnosis and thus might have been infected with other diseases instead were excluded. Moreover, we included patients from different municipalities in the state of Pernambuco, which provided us with a more heterogeneous dataset, as well as more representative and less biased sample selection.

Essentially, the main limitation of the study related to patient admission, which could be either to isolation wards or to intensive care units. Furthermore, at the time of admission via an emergency department, the notification sheet might not have been completely filled out. This would be due to high demand (several patients arriving hourly), insufficiency of medical personnel and presence of severe cases that required more attention. Additionally, data entry done from multiple locations by many different professionals would lead to inherent contrasts regarding the use of medical terms and descriptions, which would also result in heterogeneity of form-filling. Thus, it was sometimes impossible to obtain complete and accurate medical histories, including information about underlying diseases and a more detailed description of symptoms. Nonetheless, we believe that for healthcare decision-makers and medical researchers, a description of the Brazilian framework of the current pandemic is of utmost importance, in order to understand more specifically the scenario in this country.

## CONCLUSION

The novel coronavirus has been dramatically affecting developing countries like Brazil. In this country, the disease has been shown to have a broad range of symptoms and severity, including common symptoms such as coughing, fever, dyspnea and sore throat. Given the overall all-cause mortality rate of 8.06%, it is important that preventive non-pharmacological interventions should be endorsed by healthcare authorities until such time that a safe and universally available vaccine has been produced. In view of the statistical difference between patients who progressed to death and those who recovered, regarding the presence of dyspnea or respiratory distress with oxygen saturation < 95% and fever, medical providers should consider the presence of these conditions to be important prognostic factors.

We emphasize the importance of mandatory reporting systems in terms of enabling better understanding of the distribution and evolution of infectious diseases in Brazil. We therefore recommend that better and more complete investigation of medical histories and better reporting should be implemented in medical units across the country. At the present time, researchers around the world should focus their efforts on undertaking high-quality studies to assess the effectiveness of the most-used pharmacological and non-pharmacological interventions, in addition to the multiple ongoing immunization therapy trials.
